# A sub 1 GHz ultra miniaturized folded dipole patch antenna for biomedical applications

**DOI:** 10.1038/s41598-023-36747-4

**Published:** 2023-06-19

**Authors:** Abdul Rehman Chishti, Abdul Aziz, Khaled Aljaloud, Farooq A. Tahir, Qammer H. Abbasi, Zia Ullah Khan, Rifaqat Hussain

**Affiliations:** 1 Faculty of Engineering & Technology, The Islamia University of Bahawalpur, Bahawalpur, Pakistan; 2 College of Engineering, Muzahimiyah Branch, King Saud University, P.O. Box 2454, Riyadh, 11451 Saudi Arabia; 3 School of Electrical Engineering and Computer Science, National University of Sciences and Technology (NUST), Islamabad, Pakistan; 4 James Watt School of Engineering, University of Glasgow, Glasgow, UK; 5 Department of Electronic and Electrical Engineering, The University of Sheffield, Sheffield, UK; 6 School of Electronic Engineering and Computer Science, Queen Mary University of London, London, UK

**Keywords:** Engineering, Physics

## Abstract

A miniaturized folded dipole patch antenna (FDPA) design for biomedical applications operating at sub 1 GHz (434 MHz) band is presented. Antenna is fabricated on FR-4 substrate material having dimensions of 16.40 mm $\times$ 8.60 mm $\times$ 1.52 mm (0.023$\lambda$ $\times$ 0.012$\lambda$ $\times$ 0.002$\lambda$). Indirect feed coupling is applied through two parallel strips at bottom layer of the substrate. The antenna size is reduced by 83% through lumped inductor placed at the center path of the radiating FDPA, suitable for biomedical (implantable) applications and hyperthermia. Moreover, Impedance matching is achieved without using any Balun transformer or any other complex matching network. The proposed antenna provides an impedance bandwidth of 6 MHz (431–437 MHz) below − 10 dB and a gain of − 31 dB at 434 MHz. The designed antenna is also placed on a human body model to evaluate its performance for hyperthermia through Specific Absorption Rate (SAR), Effective Field Size (EFS), and penetration depth (PD).

## Introduction

Advanced development in antenna designing urges the need to integrate antenna with biomedical applications^1^, especially for cancer treatment. The application of biomedical antenna lies in early-stage cancer detection^2,3^, glucose monitoring^4^, Cardiac pacemaker^5^, endoscopy^6^ and cancer treatment through hyperthermia.

Hyperthermia is an important application of bio-medical antennas where a certain temperature is applied for a limited duration to destroy cancer cells^7^. Hyperthermia is a non-invasive and effective cancer treatment technique followed by conventional Surgery, chemotherapy and radiotherapy, and it is considered an adjuvant technique for cancer treatment^8^. Hyperthermia has been used safely for treating previously treated tumors or regrowth of the tumor cells^9^ in which temperature is raised up to 45 $^\circ$C to treat tumor tissues. This treatment method has higher efficacy with fewer side effects in contrast with the traditional treatment methods^10^.

The frequency band used for the biomedical antenna are Medical implant communication system (MICS) at (401–406) MHz and Industrial, scientific and medical (ISM) bands at 434 MHz, 915 MHz and 2450 MHz^11,12^, along with 902.8–928 MHz frequency band^13^. The Federal Communications Commission (FCC) recently revealed the wireless medical telemetry service (WMTS) band for frequencies between 608–614 MHz and 1.395–1.432 GHz^14^. Moreover, THz frequency band is also gaining popularity with antennas designed at 0.5 to 2 THz^15^, 1–4.43 THz^16^, 3.6–7.4 THz, 8.25–10.0 THz^17^, and 430 to 750 THz^18^.

Biomedical antennas operating in low-frequency bands are more suitable for cancer treatment through hyperthermia due to higher penetration depth of electromagnetic fields at low frequencies^19^. So, 434 MHz is considered a suitable frequency band for hyperthermia^11^. However, the larger antenna size at such a lower frequency may limit its applications for developing compact, portable, or implantable devices, especially for endoscopy, where the antenna is implanted inside the capsule^20^. Therefore, the major challenge while designing the compact antenna at such a lower frequency is to miniaturize its size with suitable radiation performance^21,22^.

Small antenna size also requires a complex matching network for impedance matching because of small input resistance and large reactance at the input. Therefore, the design of a simple impedance matching configuration for a miniaturized antenna is another challenge^23^. In addition, the performance of an antenna is also influenced by its proximity to the human body for hyperthermia applications^24^, hence, the appropriate design of miniaturized antenna with stable radiation performance near the human body is also a difficult task.

There are three categories of clinical hyperthermia: local, regional and whole-body.

### Local hyperthermia

In local hyperthermia, cancer cells can be destroyed if a temperature rises up to $42\,^\circ$C for one hour. It is further sub-categorized into Intraluminal Local Hyperthermia and Interstitial Local Hyperthermia.

#### Intraluminal local hyperthermia

This method is suitable for cancer cells treatment, which exists within or near the body while the applicator is inserted near the natural openings that may include gastrointestinal (esophagus and rectum), pulmonary (trachea and bronchus)^25^, gynecological (vagina, uterus and cervix) and genitourinary (bladder and prostate).

#### Interstitial local hyperthermia

Interstitial techniques are used to treat tumors deep within the body, such as brain tumors. When a single antenna fails to attain the necessary SAR uniformity over a tumor size for treatment zones that are substantial compared to penetration depth of the field at a particular frequency, arrays of antennas are used to treat such tumors.

### Regional hyperthermia

Deep-seated tumors especially inside the abdomen and pelvis, require regional heating. Deep regional hyperthermia is performed through arrays comprising multiple antennas^26^. Cancer treatment for arms, legs, and some other body organs including the liver and lungs can be treated using regional perfusion techniques.

### Whole-body hyperthermia (WBH)

For the treatment of cancer spreading throughout the body parts, whole-body hyperthermia is considered a suitable option. All through the treatment of hyperthermia, the temperature rise is continuously monitored to ensure that desired temperature for destroying cancer tumors is achieved. For hyperthermia treatment, the successful treatment is determined by the rate at which the temperature in the body rises and the final value of temperature. SAR is calculated using the formula as^27^:

1 $$\begin{aligned} SAR = 4186 \ \textit{c} \Delta T/t, \end{aligned}$$

where *c* Specific heat constant (kcal/kg), $\Delta T$ Temperature rise in $^{\circ }$C, *t* Exposed time in seconds.

However, Eq. (1) holds valid for the assumption that the metabolic rate will not increase and there is no heat loss due to blood circulation and thermal diffusion in time *t*. SAR can also be calculated from the information of the Electrical field as^28^:

2 $$\begin{aligned} SAR = \frac{\sigma }{2\rho } |{\textbf {E}}|^2, \end{aligned}$$

where, $\sigma$ Conductivity of the tissue, $\rho$ Tissue Density, ${\textbf {E}}$ Electric Field radiated in the tissue.

Equation (2) is suitable to calculate the SAR value inside tissue with information of Electric Field. Literature has suggested several antenna designs for hyperthermia and implantable applications. However, a trade-off has to be made between antenna size and performance parameters. A folded dipole feeding technique was suggested in Ref.^29^ where an electrically small antenna (ESA) was suggested comprising of Coplanar stripline (CPS) and capacitively loaded loops (CLL) to achieve quasi-isotropic radiation pattern. Antenna dimensions were taken as 20.6 mm $\times$ 20.4 mm $\times$ 0.787 mm. The antenna is fed through coaxial feed applied at the CPS. The proposed design provides an improved gain of 6 dBi at 2.4 GHz and an improved efficiency of   90%. Although the antenna operates at 2.4 GHz (ISM Band) suitable for biomedical applications, However, authors have not discussed antenna performance with respect to biomedical applications.

Antenna for hyperthermia applications require antenna performance in terms of Specific Absorption Rate(SAR) along with Effective Field Strength (EFS) and penetration depth (PD). A microwave lens applicator was recommends for the treatment of hyperthermia applications in Ref.^30^. Antenna size was taken as 32 mm $\times$ 32 mm $\times$ 3.27 mm. However, these dimensions are not suitable for implantable devices. Moreover, a superstrate was added to the design to improve the focusing ability of the proposed antenna towards the phantom, which adds extra complexity to the design.

Another work represented in Ref.^31^, suggested a sub 1 GHz antenna designed for hyperthermia. Several shapes were tested for antenna performance that, includes a rectangular patch, bow tie, truncated bow-tie, fishtail bow-tie and spiral. Antenna dimensions for the rectangular-shaped patches were 26.5 mm $\times$ 30 mm. The SAR and EFS performance of antenna was also evaluated, however, antenna size is little larger. An EFS of 12 $\times$ 18 mm$^2$ and penetration depth of 23 mm was evaluated in Ref.^32^ for Hyperthermia applications at 2.45 GHz. Antenna dimensions were 29 $\times$ 29 mm$^2$. Moreover, the relation between Absolute SAR and depth inside muscle was also discussed. EFS was much smaller for this antenna compared to antenna dimensions.

Phased array antennas are suggested in Ref.^33^ to treat breast tumors placed around the breast to focus power inside the tower. Authors have used semi-ellipsoidal and conical-shaped phased array applicators having dimensions of 178 mm $\times$ 238 mm. Eighteen patch antennas are arranged around the breast operating at the 434 MHz. SAR of 20 W/kg and 13 W/kg for ellipsoidal and conical-shaped antennas were achieved. However, such antenna comprises large dimensions and complex antenna configuration.

Another phased array applicator for breast cancer was suggested in Ref.^34^, in which 18-element phased arrays operate at 434 MHz. Using Genetic Algorithm, 18, 15, 12 and 9 antennas were activated. The temperature of $42.42\,^\circ$C, $42.48\,^\circ$C, $42.49\,^\circ$C and $42.48\,^\circ$C were measured in the tumor for the mentioned active phased arrays. Due to high power consumption, 12 active antennas are considered suitable for clinical purposes. However, no significant information regarding SAR, PD and EFS was discussed.

Article^35^, discussed the design and fabrication of a compact, ultra-thin electromagnetic bandgap (EBG) backed antenna for the 24 GHz ISM band. The proposed antenna has a Koch fractal geometry-based bow-tie slot design, backed by a 5 $\times$ 5 element EBG structure to reduce the SAR from 50.9 W/kg to less than 6.14 W/kg suitable for human exposure. The antenna is fabricated on a flexible Rogers 5880 substrate, ensuring easy integration into wearable devices and providing solutions for WBAN applications. However, its effectiveness for hyperthermia treatment may not be discussed due to insufficient information about EFS and PD.

A low profile hexagonal-shaped microstrip patch antenna was designed using FR-4 substrate, with dimensions of 124 mm $\times$ 124 mm $\times$ 1.6 mm. The ground contains orthogonal rectangular slots. Antenna operates at 434 MHz, suitable for Hyperthermia, providing SAR of 0.2 W/kg. The major drawback of this design is the large antenna dimensions with no information related to PD and EFS^36^.

A compact meta-material-based applicator specifically designed for hyperthermia cancer treatment was proposed in Ref.^37^. The applicator was made up of a double spiral antenna, an artificial magnetic conductor, and a frequency-selective surface. The applicator includes a frequency-selective surface at the top of the double spiral antenna to direct energy toward the tumor and ensure uniform heating. The research also explored the applicator’s ability to heat deeply-seated tumors, with the outcomes demonstrating a temperature profile of 44 $^\circ$C at an input power of 2.5 W. These findings suggest that the applicator can effectively heat deeply-seated tumors. However, its larger size limits its applications in developing compact devices.

A meandered dipole and loop implantable antennas operating at 434 MHz, 1.4 GHz, and 2.4 GHz was put forward in Ref.^38^, for Gastrointestinal track impedance response. Dimensions of dipole and loop are taken as 1.76 mm $\times$ 18.34 mm and 9.1 mm $\times$ 0.83 mm, respectively. The encapsulation layer was added to the design, providing the maximum 7 MHz of frequency shifting as the capsule travelled through the track.

In Ref.^39^, multiple silicon layers are suggested to improve the SAR centralization suitable for hyperthermia applications. Antenna dimensions are taken as 1.93 mm $\times$ 124 mm $\times$ 124 mm. The major drawback of this design was the antenna size, while adding multiple layers of silicon produces complexity in the antenna design. The penetration depth of up to 5 cm was achieved while EFS was varied between 14 and 89%.

In Ref.^19^, a patch antenna with circular rings was proposed with dimensions of 130 mm $\times$ 130 mm $\times$ 2.97 mm. Several antennas were compared, including a loop, dipole, square patch and compact patch. Results indicate compact patch antenna caused 51 mm penetration depth and 9100 mm$^2$ EFS. However, this antenna has large dimensions, also, the EFS was less than the actual antenna dimensions of 16,900 mm$^2$.

An open ridged-waveguide antenna (ORWA) was proposed to achieve ultrawideband in Ref.^40^ operating between 400 and 800 MHz. The EFS of the antenna exceeds its dimensions (30 mm $\times$ 40 mm) for the mentioned frequency band with input power between 7 and 25 W while SAR of 56 to 29 W/kg was achieved suitable for Hyperthermia. However, 10 and 16 such antennas were combined in this study, making the antenna design more complex with enhanced antenna dimensions.

A microwave applicator for the treatment of gynecological cancers was suggested in Ref.^28^ at 434 MHz. PD of 12 mm while EFS of 180 mm$^2$ was achieved. Applicator length and diameter are taken as 40 mm and 50 mm, respectively. However, this antenna provided low EFS with large antenna dimensions.

The ISM band of 2.4 GHz is also frequently used for biomedical antenna design. In Ref.^30^, lens applicator with dimensions of 32 $\times$ 32 $\times$ 3.27 mm$^3$ was fabricated on RT-Duroid substrate ($\varepsilon _r$ = 2.2). The antenna was placed at a distance from three-layer phantom comprising skin, fat and muscles. EFS of 56 mm $\times$ 58 mm was achieved while the SAR of 4 W/kg and PD of 33 mm were achieved. However, superstrate adds complexity to the design.

For Breast cancer hyperthermia, a microstrip applicator designed on RO 4350 ($\varepsilon _r$ = 3.48) substrate was proposed in Ref.^41^, at 915 MHz and 2450 MHz. Results indicated that 915 MHz was suitable for stage 1, 2 and 3 cancer, while 2450 MHz was only able to perform well for early-stage cancer. Antenna dimensions at 915 MHz and 2450 MHz are taken as 87.66 mm $\times$ 109.46 mm and 32.57 mm $\times$ 40.88 mm. For early-stage cancer detection, the PD of 24 mm and 27 mm, and SAR of 0.6 W/kg and 16.2 W/kg were achieved at 915 MHz and 2450 MHz, respectively. For stage 1, PD of 63 mm and SAR of 1.21 W/kg was achieved at the 915 MHz band, while at 2450 MHz Electromagnetic waves failed to enter the cancer cell region. For stage 2 and stage 3, the measurements were also mentioned against the PD and SAR.

To achieve a smaller size antenna at lower frequency band multiple lumped elements were used in the folded dipole patch antenna in Ref.^23^. The antenna size was taken as 38 mm $\times$ 0.5 mm. Increasing the Inductance shifts the resonance below the desired resonance frequency, while a smaller capacitance value was used for fine tuning of the resonance frequency. The major disadvantage of this design is still the large antenna size, complex parametric analysis of lumped elements and Balun transformation to achieve desired impedance matching.

Literature suggests that designing a smaller size antenna at sub 1 GHz is a challenging task. The miniaturized antenna can be implanted inside the skin or body and plays a vital role in bio-medial applications. Moreover, improved impedance matching along with reasonable PD, SAR and EFS is also required in miniaturized antennas suitable for hyperthermia applications.

In this work, a novel folded dipole patch antenna (FDPA) is proposed. Antenna operates at 434 MHz where miniaturization is achieved through a novel single lumped element scheme as compared to conventional multiple inductors and capacitors. A simplified design is proposed without any addition of Balun transformer or complex matching network. The EFS and penetration depth results exhibited that the proposed antenna is suitable for hyperthermia and implantable biomedical application. FDPA without lumped element produces a resonance notch at 2.65 GHz. The size reduction through lumped inductor has shifted the resonance notch at 434 MHz band, which is about 83% reduction in the size of the antenna.

The manuscript is organized as follows. “Design details” section describes the antenna design, where the antenna geometry, design procedure, and parametric analysis are discussed in detail. Results and discussion is presented in “Results and discussion” section, comprising of Specific Absorption Rate (SAR), Penetration depth (PD) and Effective Field Strength (EFS). SAR for various orientations of the antenna plane with respect to the phantom plane and the relationship of antenna distance from the phantom is discussed. Finally, the conclusion of this proposed work is presented in “Conclusion” section.

**Figure 1:**
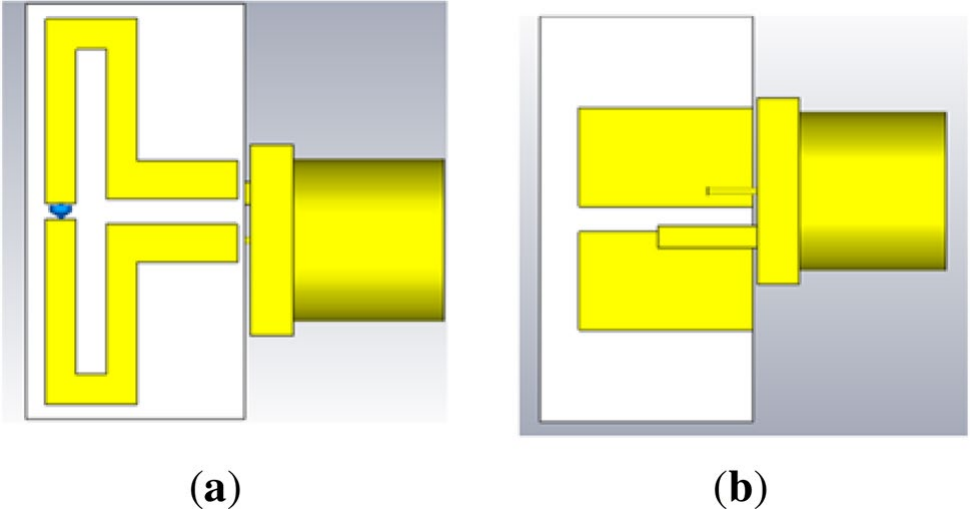
Proposed FDPA (**a**) top view, (**b**) bottom view.

**Table 1:** Optimized parameters of the proposed folded dipole antenna.

Parameter	Values (mm)	Parameter	Values (mm)
$L_{s}$	16.40	$W_{s}$	8.60
$L_{1}$	15.2	$d_{1}$	0.60
$L_{2}$	3.60	$d_{2}$	0.60
$L_{3}$	5.15	$d_{3}$	0.75
$L_{4}$	5.60	$d_{4}$	1.0
$L_{5}$	4.40	$d_{5}$	1.0
$L_{6}$	7.0	$d_{6}$	3.3
$w_{1}$	1.50	$h_{s}$	1.52

**Figure 2:**
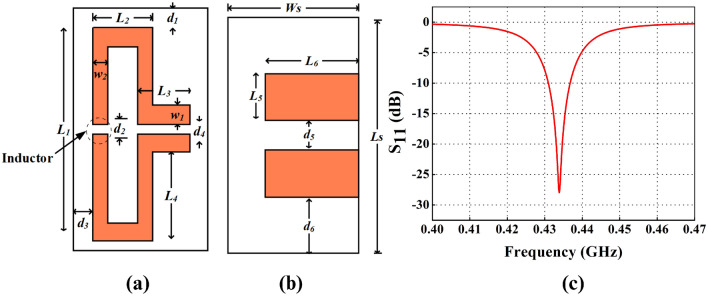
Proposed FDPA (**a**) top view dimensions, (**b**) bottom view dimensions, (**c**) return loss ($S_{11}$) of the proposed FDPA.

**Figure 3:**
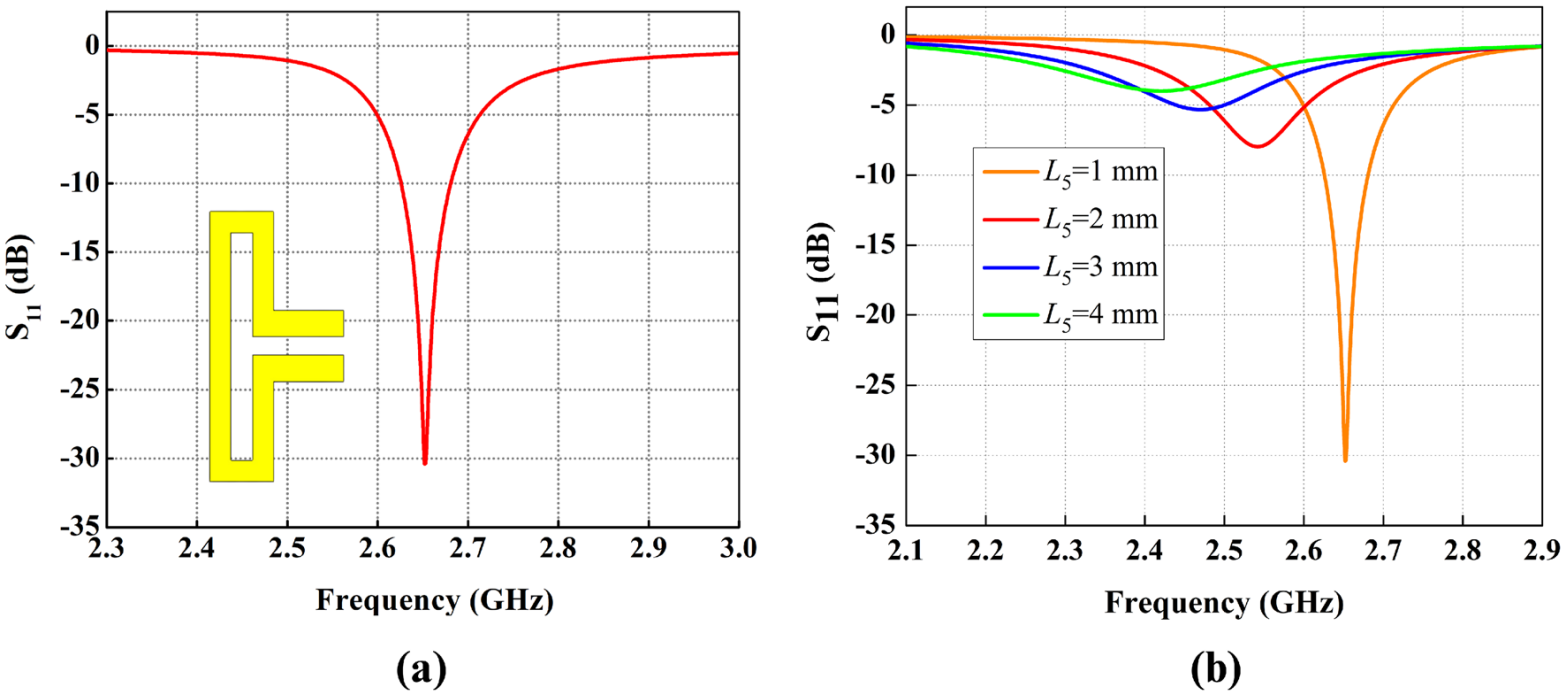
Reflection coefficient ($S_{11}$) of FDPA (**a**) without lumped element, (**b**) variations in $L_5$ without lumped element.

## Design details

This section comprises the geometry and optimized dimensions of FDPA. A parametric sweep is applied to achieve the optimized antenna performance.

### Antenna geometry

The proposed FDPA design comprises of folded dipole patch at the top layer of substrate material while two parallel strips at bottom layer of the substrate are excited through coaxial feed as illustrated in Fig. 1. Antenna is fabricated on a 16.4 mm $\times$ 8.6 mm (0.023 $\lambda$ $\times$ 0.012 $\lambda$) FR-4 substrate of 1.52 mm thickness.

An inductor (lumped element) is added to the front layer of FDPA. Several positions are changed for placing the inductor in FDPA. However, there is no significant change noticed due to the variation in inductor position, hence the inductor is added at the centre of the left length of the patch. Moreover, the gap for this inductor is kept around 1 mm. An inductor value of 200 nH with 603 package is considered suitable for this design as it can be easily soldered to this small patch width of FDPA strip. Self-resonant frequency (SRF) of the inductor should be always greater than the resonance frequency of the antenna. As the final resonance occurs at 434 MHz therefore, the inductor package chosen comprises SRF greater than 434 MHz, which in this case is taken around 800 MHz with a package length 1.2 mm suitable for ESA. Antenna dimensions are summarized in Table 1, while parameters are labelled in Fig. 2a,b.

The optimized return loss curve for the proposed FDPA is shown in Fig. 2c, it is achieved by using the dimensions mentioned in Table 1. The antenna resonates at 434 MHz with improved impedance matching without adding any complex matching circuitry.

### Antenna development steps

Initially, coaxial feed is applied to the parallel strips at bottom of the substrate layer, while no lumped component is added to the FDPA. Here, the position of the feed matters. Antenna resonates at 2.65 GHz band with $L_5$ = 1 mm. The reflection coefficient curve is shown in Fig. 3a.

**Figure 4:**
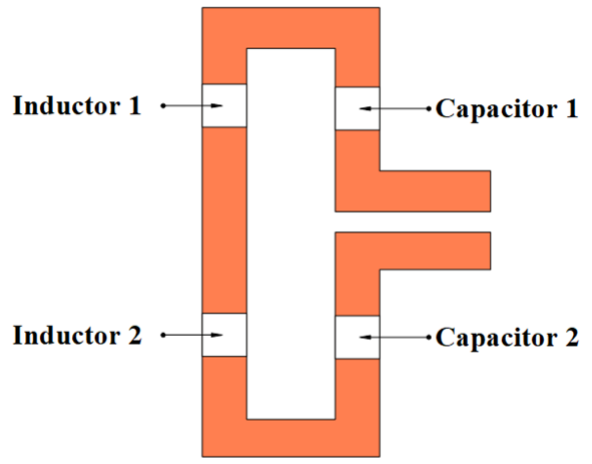
Antenna configuration with multiple lumped elements.

**Figure 5:**
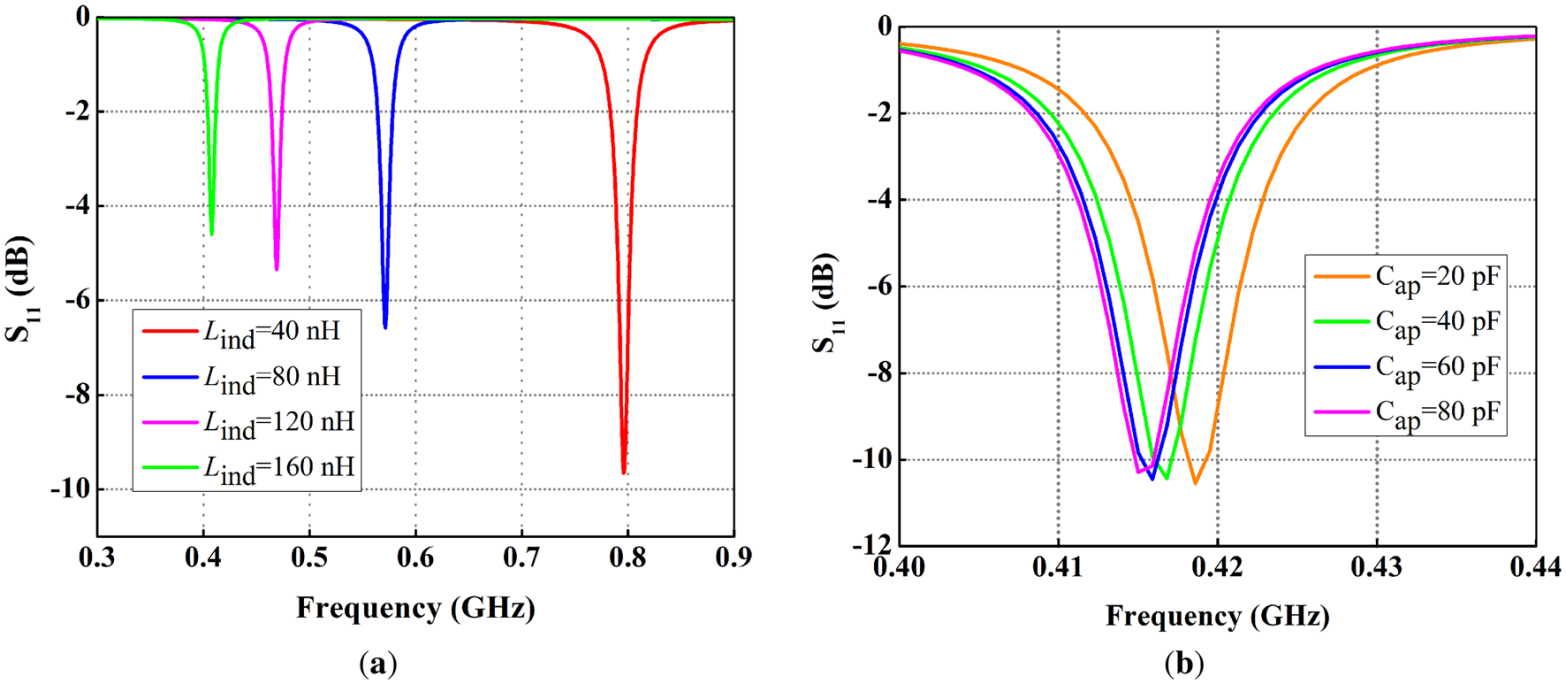
Effect of variations in lumped element values on reflection coefficient ($S_{11}$), (**a**) variation in inductance, (**b**) variation in capacitance.

**Figure 6:**
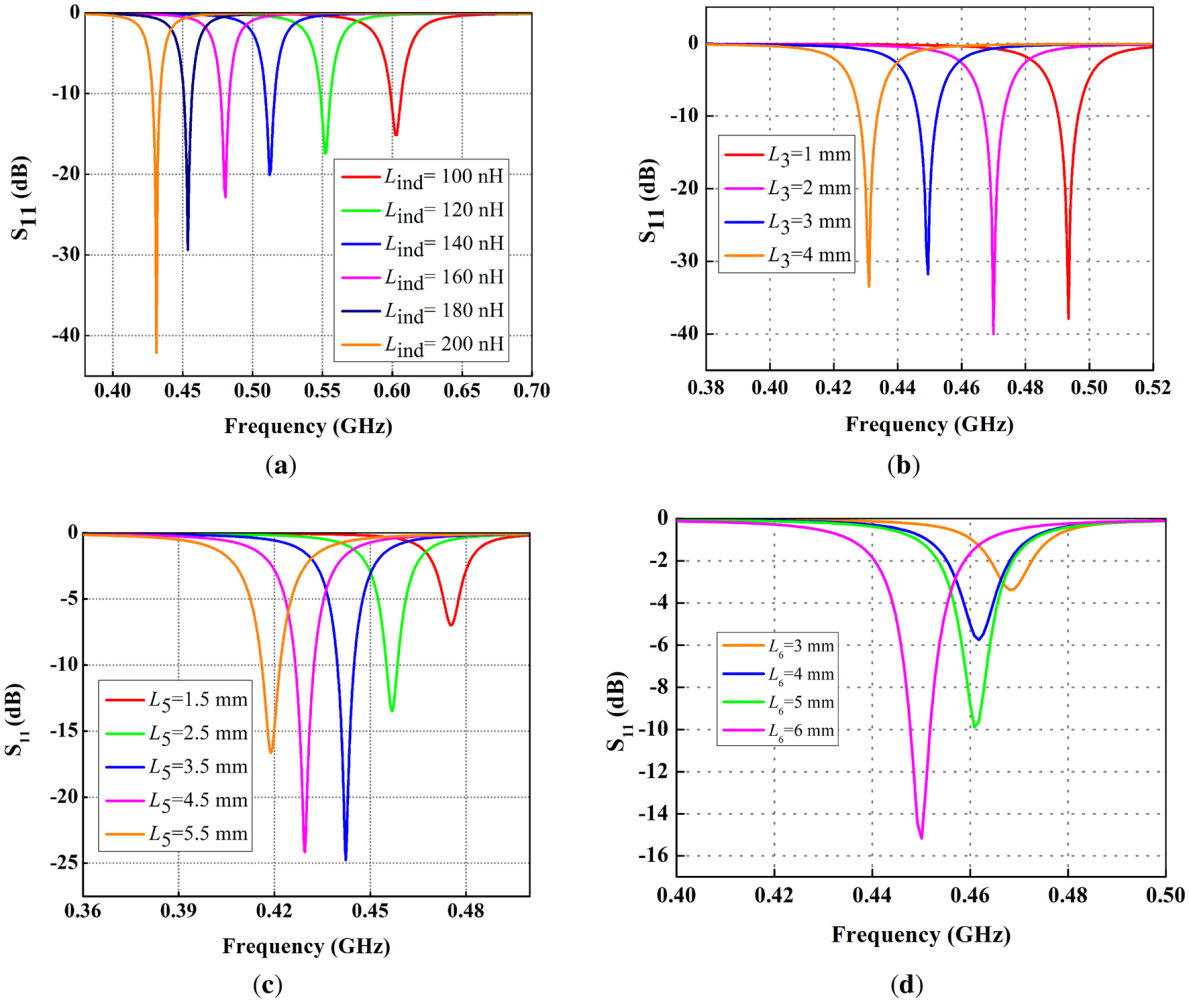
Parametric analysis of FDPA, (**a**) variation in $L_{ind}$, (**b**) variation in $L_3$, (**c**) variation in $L_5$ (**d**) variation in $L_6$.

Applying parametric sweep to the back parallel strip length $L_5$ of the inductor, the variation in the impedance matching of the antenna can be noticed as illustrated in Fig. 3b. Reflection coefficient results indicate that increasing the length of $L_5$ from 1 to 4 mm, deteriorates the impedance matching and return loss changes from − 30 to − 5 dB, and also slight frequency shift is observed from 2.67 to 2.45 GHz; however, at 2.65 GHz the best impedance matching with a return loss of − 30 dB is achieved.

In the second step, four lumped elements are added in the design comprising of two inductors and two capacitors, to shift the resonance frequency to sub 1 GHz band, while the feed is provided to the parallel strips placed at the bottom layer of the substrate. The position of lumped elements on the top layer of the folded dipole patch antenna is illustrated in Fig. 4. A left strip of the folded dipole contains two inductors, labeled inductor 1 and inductor 2, while capacitor 1 and capacitor 2 are connected to the right portion of the strip. The addition of these elements causes shift in resonance frequency in sub 1 GHz band. The simulation time of the CST studio also varies with the change in the inductance and capacitance values.

Figure 5a, shows that a higher value of inductance shifts the resonance below the required frequency, while the small value of capacitance can be used for fine tuning of the required resonance frequency as shown in Fig. 5b. However, the use of multiple lumped elements adds complexity to the antenna design, while choosing their values through parametric sweep is also tedious. Therefore, to reduce the complexity of multiple lumped elements, the single inductor is introduced in the design, and careful selection of inductor value through parametric analysis resulted in frequency shift from 2.65 to 0.434 GHz.

### Effect of variation in $L_{ind}$ on $S_{11}$

To shift the impedance to sub 1 GHz, an inductor is introduced in the design of FDPA. The position of the inductor on the vertical length of the folded dipole does not create any difference in the shifting of the resonance frequency. Figure 6a below, demonstrates the effect on Reflection coefficient ($S_{11}$) with variations in inductance ($L_{ind}$). Inductor variation is taken between 100 and 200 nH. Applying parametric optimization to the $L_{ind}$ indicates that with an increase in $L_{ind}$, the $S_{11}$ shifts to a lower frequency as illustrated in Fig. 6a. The lowest value of 100 nH results in resonance at 0.6 GHz, while the inductor value of 202 nH causes a shift in resonance frequency from 2.65 to 0.434 GHz, which covers the ISM band suitable for biomedical applications.

### Effect of variation in ${L_{3}}$ on $S_{11}$

Figure 6b shows the effect on the reflection coefficient for variation in arm length $L_3$ of folded dipole, where the arm length is varied between 1 and 4 mm. Variation in the length of folded dipole arm $L_3$ increases or decreases the electrical length of the radiating patch. A decrease in length shifts the resonance at high frequency, while an increase in the arm length $L_3$ shifts the frequency towards lower value.

### Effect of variation in back strip width ($L_5$) on $S_{11}$

Variation in $L_5$ results in shifting of the resonance frequency as reflected in Fig. 6c, and it also effect impedance matching. So, $L_5$ is used for fine tuning of resonance frequency and impedance matching.

### Effect of variation in back strip length ($L_6$) on $S_{11}$

The variations in the length of parallel strips ($L_6$) have almost similar effect to that of the width $L_5$ of back strips. Improved impedance matching is achieved at $L_6$ = 6 mm, indicating that increasing the length will improve the impedance matching, however, a slight frequency shift is observed by varying $L_6$ as illustrated in Fig. 6d.

## Results and discussion

The final design represents the antenna resonance shifted from 2.65 GHz to 434 MHz (sub 1 GHz band). The appropriate value of the Lumped element (inductor) is chosen after various parametric sweeps. A bandwidth of 6 MHz (431–437 MHz) is achieved in this design.

**Figure 7:**
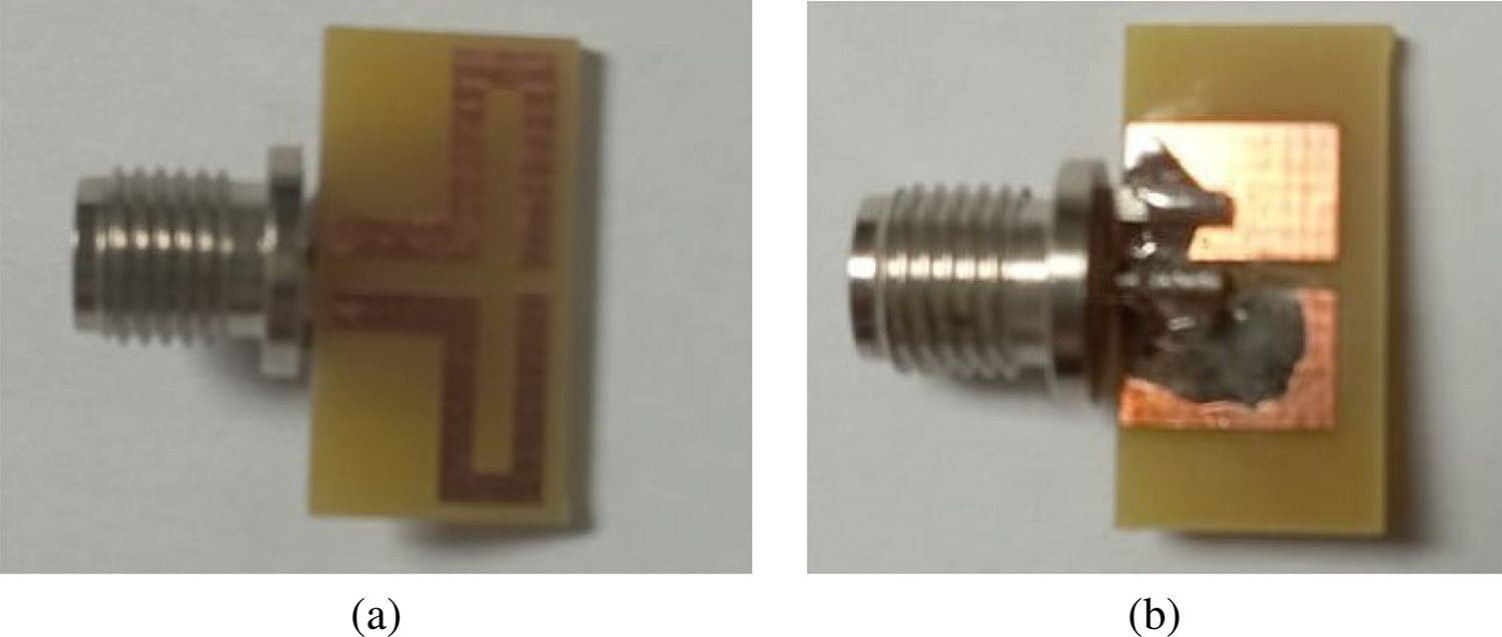
Prototype of the proposed FDPA (**a**) top layer, (**b**) bottom layer.

**Figure 8:**
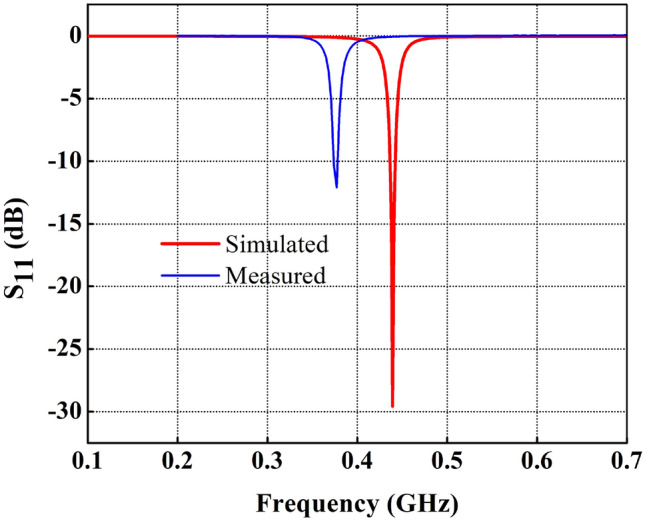
Comparison of measured and simulated reflection coefficients of FDPA.

**Figure 9:**
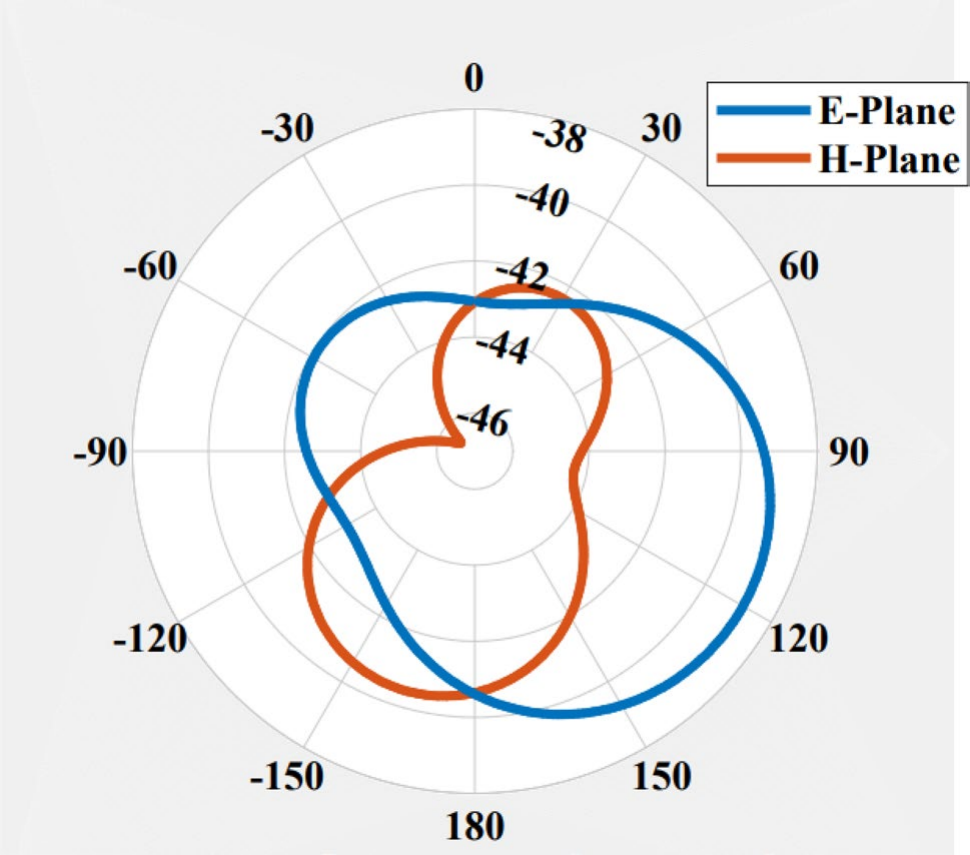
Radiation pattern of FDPA at 434 MHz in E and H planes.

**Figure 10:**
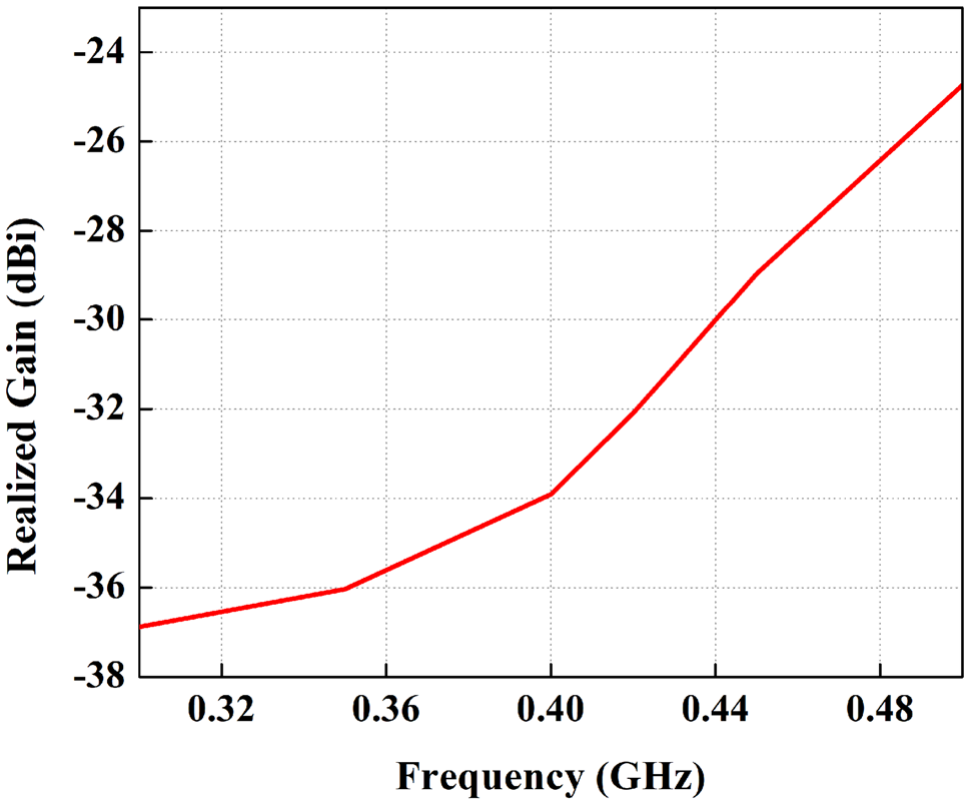
Maximum gain over frequency for the proposed FDPA.

The fabricated antenna is presented in Fig. 7a,b, where the top and bottom layer of the prototype is shown. A comparison of simulated and measured return loss is illustrated in Fig. 8. A slight shift in resonance frequency towards lower frequency with a little higher return loss is observed. This little deviation is may be due to parasitic resistance and capacitance effect of the inductor (lumped element), which may not be considered precisely in the simulation environment, and such an effect may lower the resonance frequency of the proposed antenna. Additionally, as the size of the antenna is very small, so, imperfect soldering of SMA connector may also vary the effective width of the parallel strips and hence impedance matching of the proposed antenna which may increase a little the return loss, however, it is still lower than − 10 dB in the mentioned frequency band.

The radiation patterns for the proposed antenna in both E and H planes are illustrated in Fig. 9. The maximum gain over the frequency response of the proposed FDPA is presented in Fig. 10. Gain is taken over the range of frequencies between 0.3 and 0.5 GHz. The antenna gain at 434 MHz is − 31 dBi. The results indicate that the antenna gain is increased with an increase in frequency.

### SAR, PD and EFS measurements

For measurements of SAR, PD and EFS, the proposed FDPA is placed near the three-layer phantom comprising skin, fat and muscle layer. Each layer has its own permittivity, conductivity and density as mentioned in Table 2 depending on the distance of the antenna from these layers, also the thickness of these layers effects the SAR, PD and EFS.

**Table 2:** Parameters of three-layer phantom^39^.

Layer name	Thickness (mm)	Permittivity ($\varepsilon _r$)	Conductivity (S/m)	Density (kg/m$^3$)
Skin	1	46.05	0.709	3150
Fat	2	5.56	0.041	916
Muscle	25	56.8	0.805	1046

**Figure 11:**
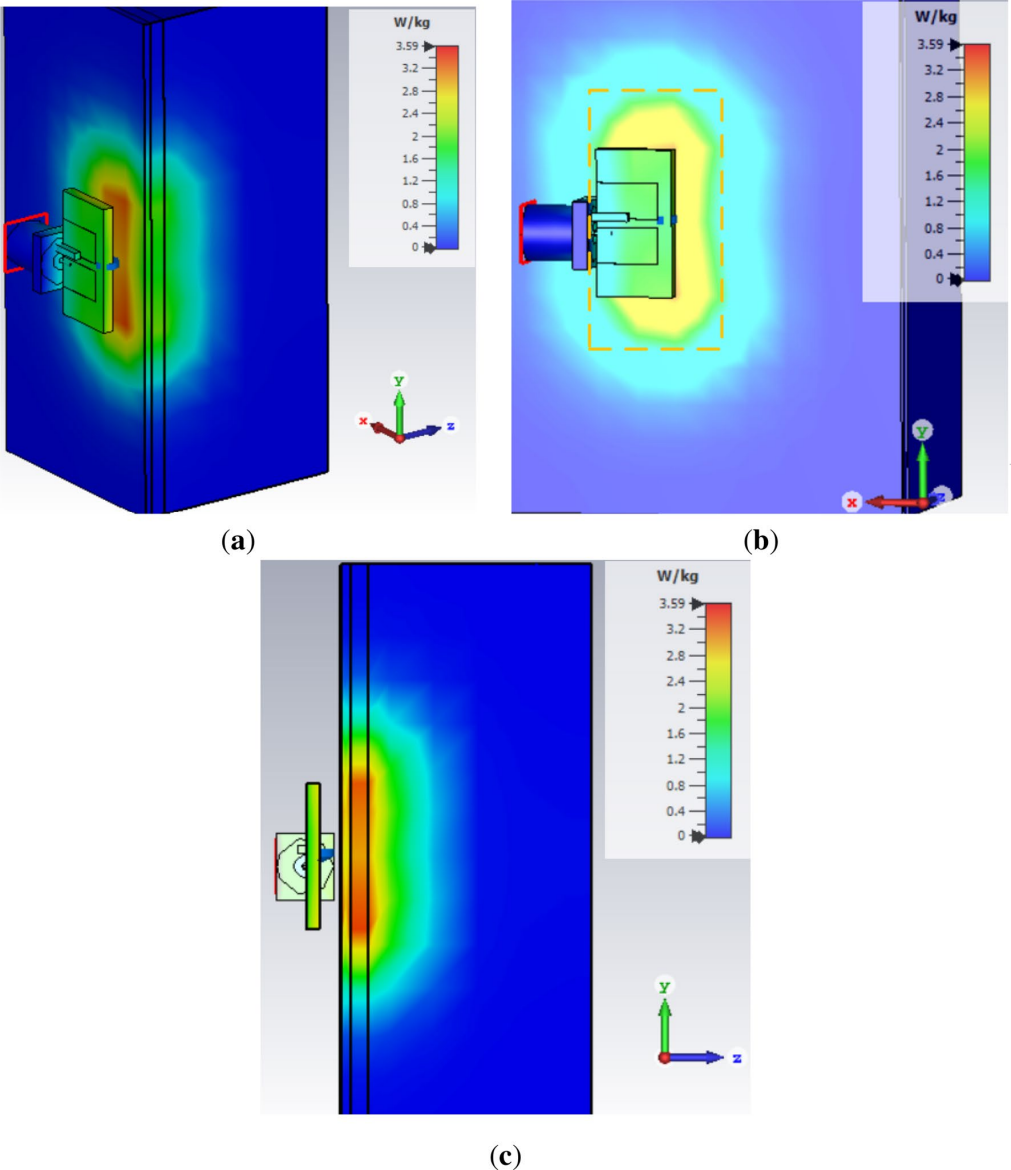
Measurements of SAR, EFS and PD on 3-layer phantom of human body (**a**) SAR, (**b**) EFS and (**c**) PD.

Initially, the antenna is placed at a 3 mm distance from the three-layer phantom comprising of skin, fat, and muscles having thicknesses of 1 mm, 2 mm and 25 mm respectively. SAR measured is 3.59 W/kg at 0.5 W input power. EFS of 17 mm $\times$ 22 mm, measured in the area which is 50% enclosed inside the phantom. Figure 11a shows the maximum SAR value when the antenna is placed at 3 mm distance from the three-layer phantom, while the dotted rectangle in Fig.  11b illustrates the EFS occupying the 50% contour inside the phantom. Moreover, 1/$\textit{e}^2$ of SAR represents the penetration depth (PD) of 11.5 mm as given in Fig. 11c.

### Effect of antenna orientation on SAR

**Figure 12:**
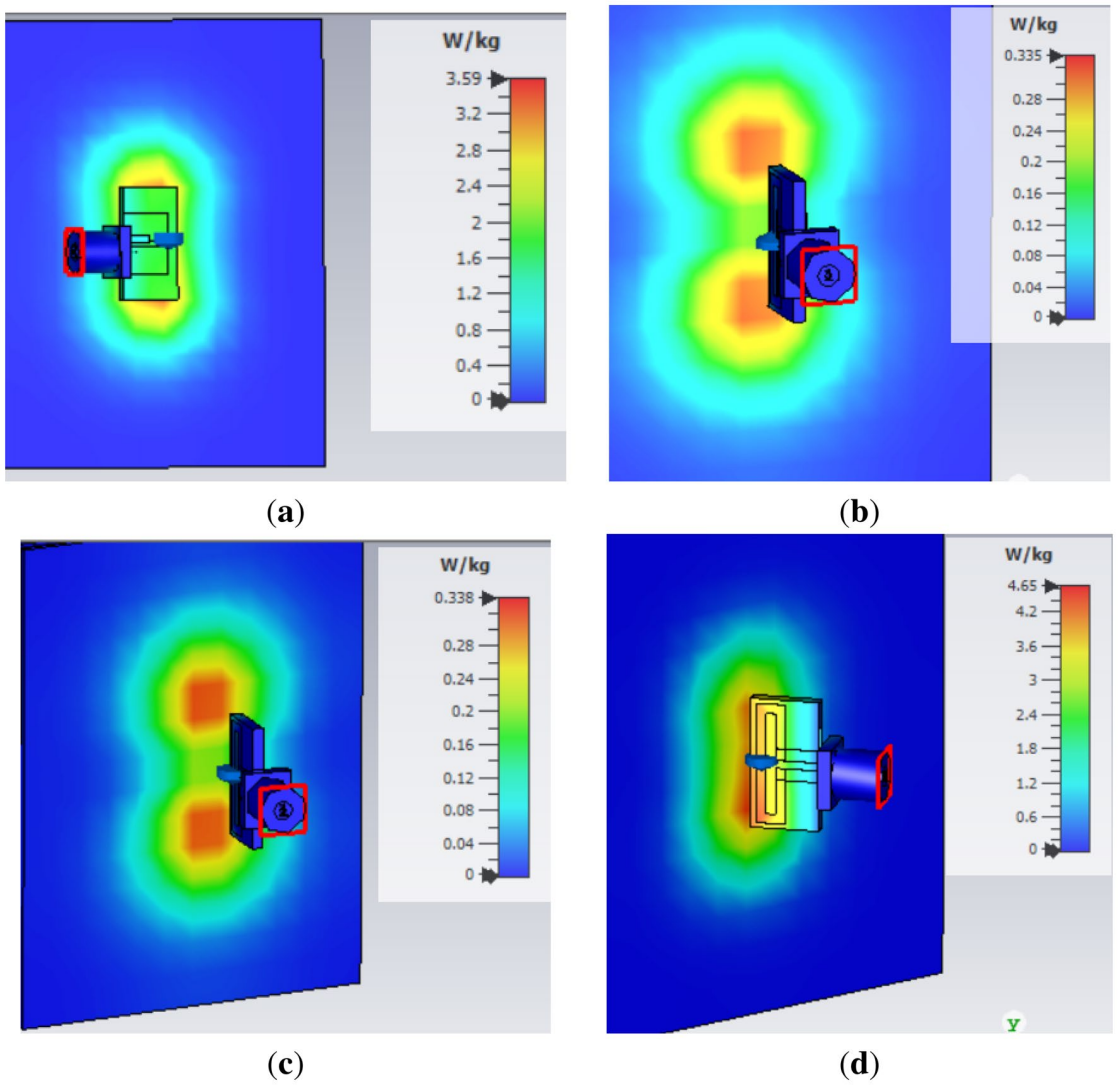
SAR for various orientations of antenna plane with respect to the phantom plane. (**a**) Parallel to the phantom plane. (**b**) Normal to the phantom plane. (**c**) 80$^\circ$ offset from the phantom plane. (**d**) 130$^\circ$ offset from the phantom plane.

SAR for various orientations of antenna plane with respect to the phantom plane is presented in Fig. 12. First, the antenna plane is aligned parallel to the phantom plane as illustrated in Fig. 12a, this orientation causes SAR value of 3.59 W/kg on phantom. In Fig. 12b, the antenna plane is normal to the phantom plane, which excites SAR value 0.335 W/kg on phantom. Subsequently, the antenna plane is oriented at angles of 80$^\circ$ and 130$^\circ$ from the phantom plane, which results in SAR values of 0.338 W/kg and 4.65 W/kg as shown in Fig. 12c,d, respectively.

**Figure 13:**
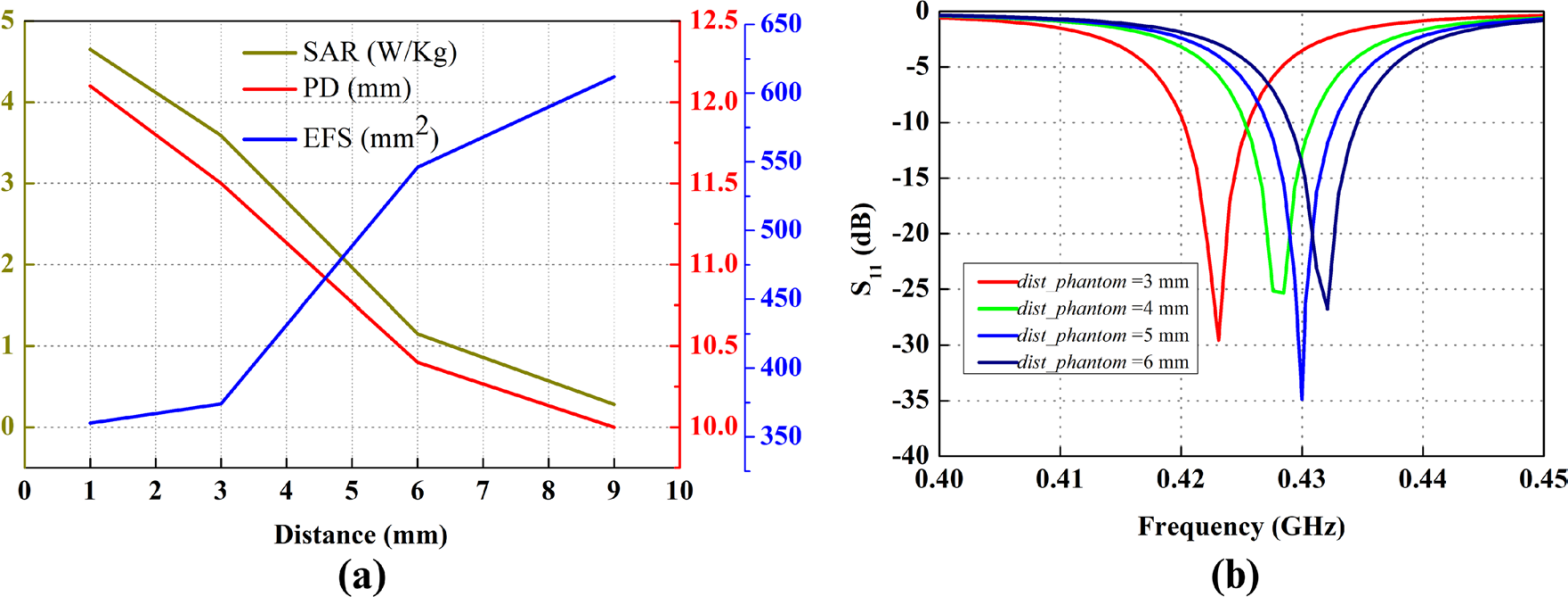
Effect of distance variation between FDPA and human phantom for (**a**) SAR, PD and EFS, (**b**) $S_{11}$.

As the antenna contains no ground plane, therefore maximum radiation direction of the antenna is not along broadside, instead, the maximum radiation is noticed at 130$^\circ$ from the phantom plane, hence the maximum SAR is noticed at 130$^\circ$. The variations in the distance between the antenna and penetration depth resulted in altered SAR, EFS and PD values. As the distance increases, the SAR and PD decreases while EFS increases. Similar measurements are made for antennae placed at 3 mm, 6 mm and 9 mm from the phantom and above mentioned quantities (EFS, PD and SAR) are measured. Their relationship graph is represented in Fig. 13. When the antenna is placed at 1 mm distance from the phantom, a maximum SAR of 4.65 W/kg is achieved whereas, the PD and EFS of 12 mm and 360 mm$^2$ is noticed as represented in Fig. 13a.

The shift in resonance frequency also depends on the distance of the antenna from the body (phantom). For the case of FDPA, a minor shift is observed between 0.42 and 0.43 GHz, as antenna distance is varied between 3 and 6 mm, as indicated in Fig. 13b. This shift is observed due to different dielectric constants of the 3-layers phantom. The more FDPA is placed closer to the phantom, the dielectric material’s effect of the 3-layer phantom (skin, fat and muscles) is more dominant and the return loss curve shift towards 0.42 GHz is observed, while at little larger distance i.e. 6 mm, the impact of the phantom on antenna performance is negligible.

## Conclusion

This work presents a miniaturized FDPA suitable for hyperthermia and implantable biomedical applications. The proposed antenna is electrically small, where impedance matching is achieved without any complex matching circuit or Balun transformer. Initially, the antenna resonates at 2.65 GHz while changing the position of the feed point and adding the lumped element to the folded dipole patch; it resonates in sub 1 GHz band at 434 MHz. Later the SAR, EFS and PD of the antenna in 3-layer human phantom is also calculated for a distance of 3 mm, 6 mm and 9 mm between the FDPA and the phantom. The proposed FDPA provides good performance for SAR, EFS and PD with the additional advantages of simple design and miniaturized size.

## Data Availability

All data required to evaluate the findings of this work is available in the presented paper. Additional data related to this work may be requested from the corresponding author.
